# Supplementary material for: Medicinal Values and Potential Risks Evaluation of Ginkgo biloba Leaf Extract (GBE) Drinks Made from the Leaves in Autumn as Dietary Supplements
Source: Molecules. 2022 Nov 2;27(21):7479. doi: 10.3390/molecules27217479 (PMC9658157; doi:10.3390/molecules27217479)
Supplement: Supplementary file 1 [file molecules-27-07479-s001.zip › molecules-1895480-supplementary.pdf]

Supplementary Materials

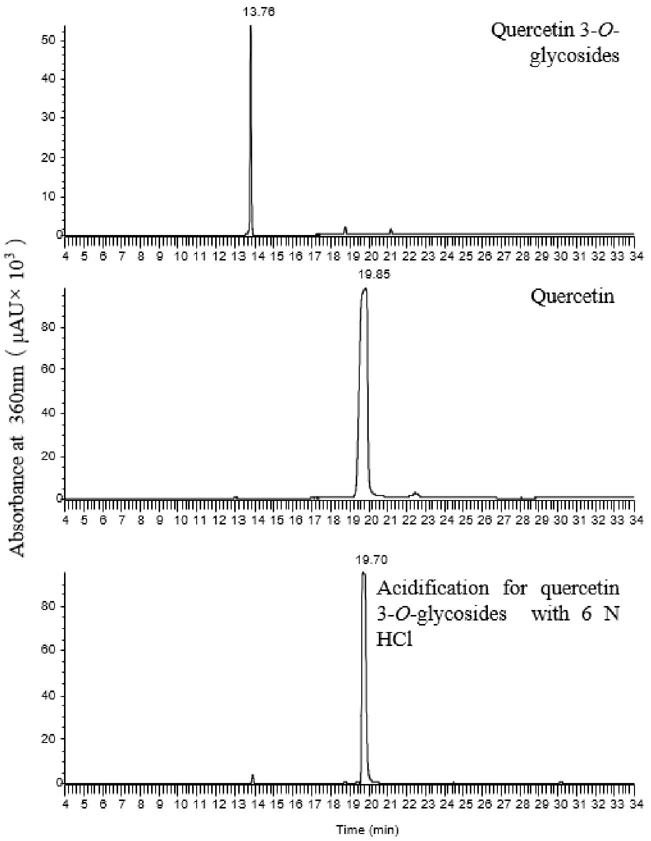

Figure S1. The dependability confirmation of acidification methods for flavonol aglycones analyses. The HPLC-MS/MS chromatograms of authentic quercetin 3-*O*-glycoside (upper panel), quercetin (middle panel) and acidification of quercetin 3-*O*-glycosides with 6 N HCl (lower panel).

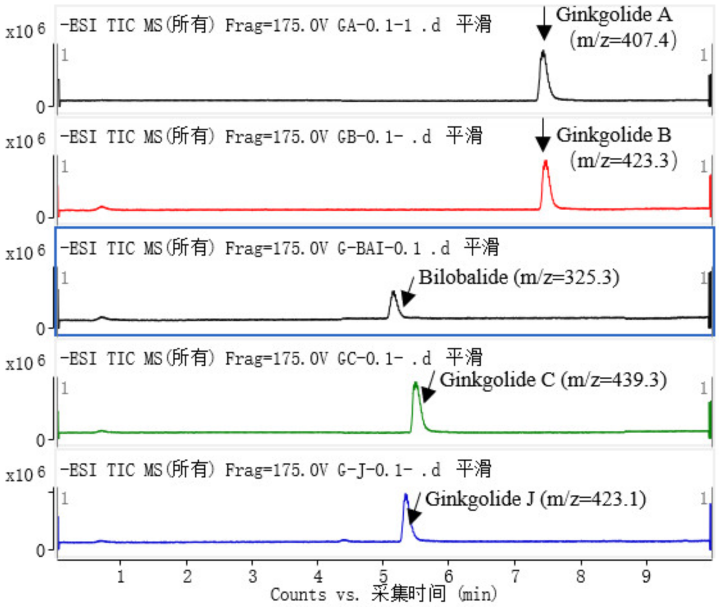

Figure S2. MS ion-current amplifier chromatograms of the TTLs authentic substrates (0.1nmol) at negative mode in ginkgo wine and tea from the GL & YL powder through UHPLC–QTOF-MS. The arrows indicated the specific response signal calculated according to the authentic substrates.

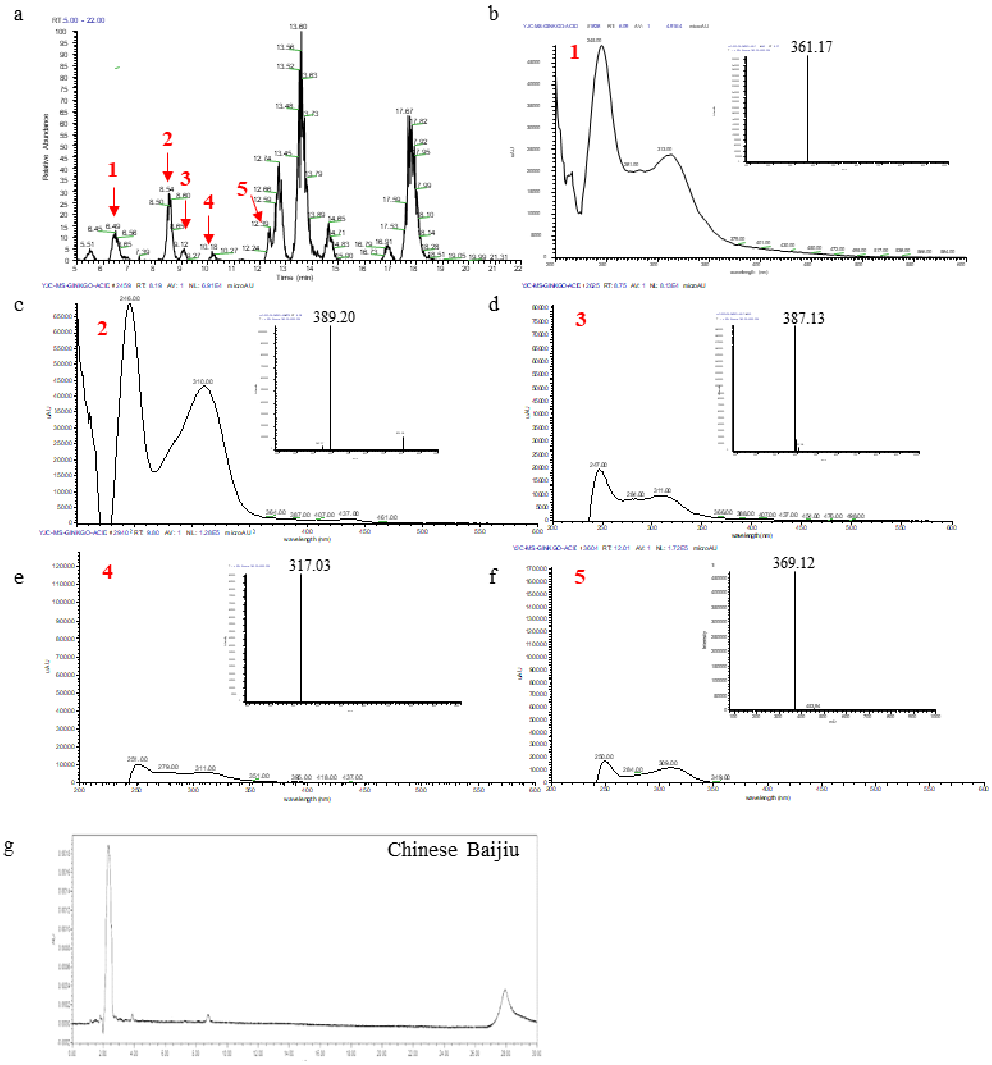

Figure S3. The mass spectra and UV spectrum of ginkgo wine from YL for the determination of total ginkgolic acids.

(a), the MS ion-current amplifier chromatograms of ginkgo wine from YL.

(b-f), the UV spectrum and mass spectra of the deduced newly-identified types of ginkgolic acids of 1-5 marked in **(a)**.

(g), the HPLC chromatograms of Chinese Baijiu as the blank control of the ginkgolic acid determination.

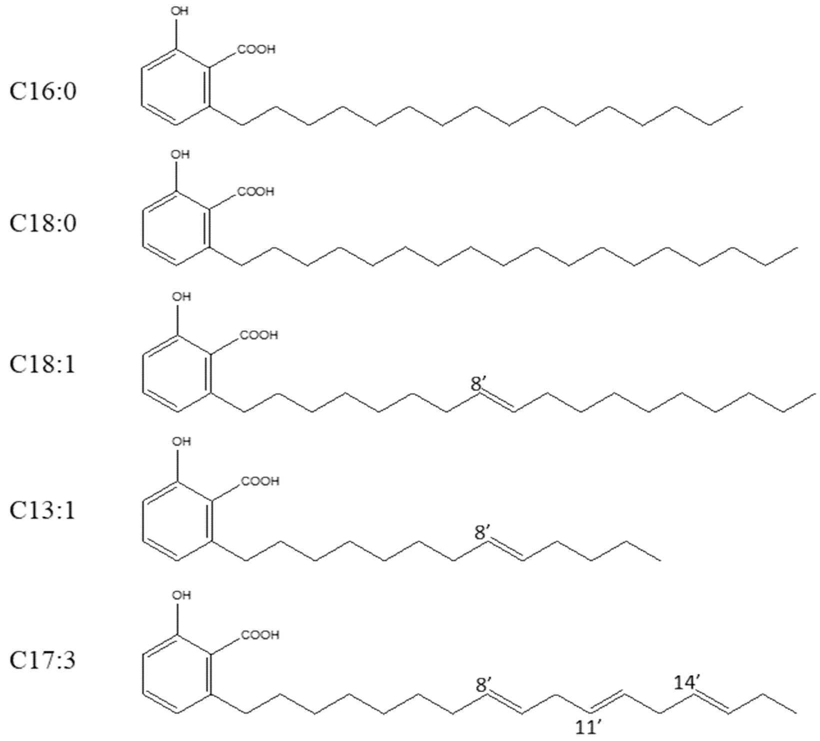

Figure S4. The deduced chemical constructions of the five newly-identified ginkgolic acids. The chemical constructions were deduced according to the description before [2, 32] and information in similar compounds identified in other species [33]. The specific ginkgolic acids chemical structures were drawn by ChemDraw Ultra 8.0.
